# Impact of Sustained Transforming Growth Factor-β Receptor Inhibition on Chromatin Accessibility and Gene Expression in Cultured Human Endometrial MSC

**DOI:** 10.3389/fcell.2020.567610

**Published:** 2020-09-01

**Authors:** Raffaella Lucciola, Pavle Vrljicak, Shanti Gurung, Caitlin Filby, Saeedeh Darzi, Joanne Muter, Sascha Ott, Jan J. Brosens, Caroline E. Gargett

**Affiliations:** ^1^The Ritchie Centre, Hudson Institute of Medical Research, Melbourne, VIC, Australia; ^2^Department of Obstetrics and Gynaecology, Monash University, Melbourne, VIC, Australia; ^3^Division of Biomedical Sciences, Warwick Medical School, University of Warwick, Coventry, United Kingdom; ^4^Tommy’s National Centre for Miscarriage Research, Warwick Medical School, University Hospitals Coventry and Warwickshire National Health Service Trust, Coventry, United Kingdom

**Keywords:** endometrium, mesenchymal stem cell, human, chromatin, gene regulatory networks, transforming growth factor receptor beta, retinoic acid receptor beta

## Abstract

Endometrial mesenchymal stem cells (eMSC) drive the extraordinary regenerative capacity of the human endometrium. Clinical application of eMSC for therapeutic purposes is hampered by spontaneous differentiation and cellular senescence upon large-scale expansion *in vitro*. A83-01, a selective transforming growth factor-β receptor (TGFβ-R) inhibitor, promotes expansion of eMSC in culture by blocking differentiation and senescence, but the underlying mechanisms are incompletely understood. In this study, we combined RNA-seq and ATAC-seq to study the impact of sustained TGFβ-R inhibition on gene expression and chromatin architecture of eMSC. Treatment of primary eMSC with A83-01 for 5 weeks resulted in differential expression of 1,463 genes. Gene ontology analysis showed enrichment of genes implicated in cell growth whereas extracellular matrix genes and genes involved in cell fate commitment were downregulated. ATAC-seq analysis demonstrated that sustained TGFβ-R inhibition results in opening and closure of 3,555 and 2,412 chromatin loci, respectively. Motif analysis revealed marked enrichment of retinoic acid receptor (RAR) binding sites, which was paralleled by the induction of *RARB*, encoding retinoic acid receptor beta (RARβ). Selective RARβ inhibition attenuated proliferation and clonogenicity of A83-01 treated eMSC. Taken together, our study provides new insights into the gene networks and genome-wide chromatin changes that underpin maintenance of an undifferentiated phenotype of eMSC in prolonged culture.

## Introduction

The human endometrium is a highly dynamic tissue that generates 4–10 mm of mucosa in each menstrual cycle (Jabbour et al., 2006). This extraordinary regenerative capacity is mediated by resident epithelial progenitors and mesenchymal stem/stromal cells (MSC) (Chan et al., 2004; Gargett et al., 2016). Cultured endometrial MSC (eMSC) are clonogenic, highly proliferative, multipotent, and express the International Society for Cellular Therapies (ISCT) surface markers (Gargett et al., 2009). Human eMSC can be purified as CD140b^+^CD146^+^ cells (Schwab and Gargett, 2007), or by means of the surface marker SUSD2 (Sushi Domain-containing 2, formerly W5C5) (Masuda et al., 2012). While eMSC also express Stro-1, this marker does not enrich for clonogenic cells (Schwab et al., 2008). eMSC further express MSCA-1 (Sobiesiak et al., 2010). These markers identify eMSC as pericytes and perivascular cells of endometrial spiral arterioles, arterioles and venules (Gargett and Masuda, 2010; Murakami et al., 2014; Lucas et al., 2016; Bozorgmehr et al., 2020). A major advantage of the endometrium over other sources of adult MSC, such as bone marrow or adipose tissue, is its accessibility. Endometrial sampling is a routine office-based procedure that does not require anesthesia (Gargett and Masuda, 2010).

Preclinical animal studies showed that eMSC are a promising cell source for treatment of gynecological disorders, including pelvic organ prolapse (Darzi et al., 2016; Gargett et al., 2019). For example, eMSC seeded or bio-printed onto meshes with biomechanical properties matching the human vagina (e.g., non-degradable polyamide/gelatin composite meshes) (Ulrich et al., 2014; Emmerson et al., 2019), or on degradable nanofibers (Mukherjee et al., 2019b; Paul et al., 2019), promote angiogenesis, collagen deposition, and cellular infiltration into biomaterials when transplanted into rodent or ovine models. They also elicit an early inflammatory response, characterized first by influx of M1 macrophages, which then switch to the M2 wound healing phenotype (Ulrich et al., 2014; Darzi et al., 2018). *In vitro*, eMSC seeded on polyamide/gelatin meshes differentiate into smooth muscle cells and fibroblasts; cell types that are important for restoring vaginal structure and function (Su et al., 2014). Hence, the endometrium is a rich source of MSC for autologous and allogenic cell-based therapies, including pelvic organ prolapse, urinary incontinence, and regeneration of scarred endometrium in women with Asherman’s syndrome (Ulrich et al., 2013; Gargett et al., 2016; Simoni and Taylor, 2018).

Clinical application of eMSC requires expansion of cells in culture (Gurung et al., 2015; Darzi et al., 2016). As is the case for MSC from other sources (Baxter et al., 2004), eMSC cultured over several passages differentiate spontaneously into endometrial stromal fibroblasts as shown by signature gene profiles and are subjected to replicative stress caused by telomere shortening (Gurung et al., 2015, 2018b; Barragan et al., 2016). Consequently, the cells lose their proliferative capacity as well as the ability to reconstitute tissue *in vivo* (Baxter et al., 2004). To overcome this impediment to the clinical translation, we and others have explored the use of small molecules to maintain the *in vivo* properties of eMSC and MSC in prolonged culture (Al-Habib et al., 2013; Gurung et al., 2015, 2018b). In particular, we focused on blocking the TGFβ receptor signaling pathway as it is one of the main pathways inducing MSC differentiation (Ng et al., 2008). A83-01 is also used to prevent spontaneous differentiation of human pluripotent stem cells (Li and Ding, 2010) and organoid cultures for a range of epithelial cell types (Sato and Clevers, 2013). We recently reported that A83-01, a selective inhibitor of TGF-β type I receptor (TGFβ-R) ALK4, 5 and 7 kinase, increases proliferation and inhibits apoptosis and senescence of cultured eMSC, thereby safeguarding their functional properties *in vitro* (Gurung et al., 2015, 2018b) and prolonging their survival *in vivo* (Gurung et al., 2018a). Further, A83-01-treated eMSC maintain multipotency, exhibit increased angiogenic activity, express a proangiogenic, antifibrotic, and immunomodulatory gene profile and enhanced secretory profile of angiogenic and immunomodulatory factors (Gurung et al., 2015, 2018b).

The mechanisms underlying the expansion of cultured eMSC in response to sustained TGFβ-R inhibition are incompletely understood. Adult stem/progenitor cells have more open chromatin than their differentiated progeny, but less than pluripotent stem cells. Further dynamic chromatin changes underpin subsequent differentiation into mesodermal lineages (Chen and Dent, 2014), including fibroblasts. Based on these observations, we hypothesized that sustained TGFβ-R inhibition leads to divergence in the chromatin landscape of cultured eMSC and activation of transcriptional regulatory circuitries that maintain the cells in a more naïve or undifferentiated state. To test this hypothesis, eMSC cultured with or without A83-01 were subjected to integrated RNA-sequencing (RNA-seq) and Assay for Transposase Accessible Chromatin through sequencing (ATAC-seq). Our findings provide new insights into the mechanisms of action of TGFβ-R inhibition that may lead to the development of more targeted pharmacological approaches for eMSC expansion, thereby enhancing their functional properties for clinical translation.

## Materials and Methods

### Endometrial Biopsies

The study was approved by the Monash Health and Monash University Human Research Ethics committees. Endometrial biopsies were obtained from seven pre-menopausal women, without endometrial pathologies, following written informed consent and according to The Declaration of Helsinki (2000) guidelines. Participant information is kept confidential and samples deidentified prior to use. None of the participants received hormonal treatment within 3 months prior to the biopsy.

### eMSC Isolation and Culture

Endometrial biopsies were processed and single-cell suspensions of eMSC obtained as described previously (Masuda et al., 2012). Briefly, finely minced endometrial tissue was enzymatically and mechanically digested in Dulbecco’s modified Eagle’s medium (DMEM/F12) supplemented with collagenase type I and DNase I (Worthington Biochemical Corporation, United States) at 37°C on a rotating MACSmix (Miltenyi Biotec, United States) for 60 min. The digested tissue was filtered through 40 μm strainers (Becton Dickinson, BD, United States) to remove gland fragments, washed, red blood cells removed using Ficoll-Paque (GE healthcare Bio-science, United States) density gradient separation at 1500 rpm for 15 min, and the interface containing stromal cells was washed in DMEM/F12/10% fetal calf serum (FCS, Invitrogen, United States) and 1% primocin (Life Technologies, United States). Single-cell suspensions were re-suspended in separation buffer (0.5% FCS/PBS), and incubated with 10 μg/ml phycoerythrin (PE)-conjugated anti-human SUSD2 (BioLegend, United States) in the dark at 4°C for 30 min. Cells were then washed, the cell pellet re-suspended in 20 μl of anti-PE magnetic-activated cell sorting (MACS) microbeads (Miltenyi Biotec) with 80 μL separation buffer and incubated in the dark at 4°C for 30 min. The cells were washed, re-suspended in separation buffer 500 μL and applied to a Miltenyi column (Miltenyi Biotech) in a magnetic field. Columns were washed three times with buffer. Magnetically labeled SUSD2^+^ eMSC were eluted with buffer and cell number was determined using Koya glasstic slides (KOVA International, United States).

eMSC were cultured in DMEM/F12 medium containing 10% FCS, 1% primocin and 2 mM glutamine (Invitrogen), supplemented with 10 ng/ml basic fibroblast growth factor (bFGF) (Peprotech, United States) and scaled-down to an in-house DMEM/F12 serum free medium (SFM) over 48 h within the first passage and incubated at 37°C in 5% CO_2__/_5% O_2_/90% N_2_, since this physiological O_2_ concentration favored eMSC expansion as described previously (Rajaraman et al., 2013), with 1 μM TGFβ-R inhibitor, A83-01 (Tocris Bioscience, United States) or vehicle control (0.01% DMSO). Cells were seeded at 5,000 cells/cm^2^ with medium changed every 48 h and passaged on days 15, 22, 29, and 36 into fibronectin-coated (10 μg/ml; BD, United States) culture flasks as described previously (Gurung et al., 2015). At each passage, cells were counted using Kova glasstic slides and cumulative cell population (total cell number) calculated by multiplying total number of cells yielded at the current passage by total number of cells yielded at the previous passage and then dividing by the number of cells seeded at the current passage, as described previously (Gargett et al., 2009). At passage 4 (i.e., 36 days in culture), untreated and A83-01-treated eMSC cultures were subjected to functional assays, and RNA- and ATAC-seq.

### Flow Cytometry

The surface phenotype of untreated and A83-01-treated eMSC was assessed by flow cytometry for three MSC markers [SUSD2, CD140b (Platelet-derived growth factor receptor β) and CD90 (Thy-1)] as described previously (Gurung et al., 2015). Cells were incubated for 1 h with 1:20 primary or matched isotype control antibodies in PBS containing 2% FCS at 4°C in dark. The primary antibodies were APC-conjugated SUSD2 (Biolegend), PE-conjugated CD140b (R&D Systems, United States) and APC-conjugated CD90 (BD Pharmingen, United States). Cells were washed with 2% FCS/PBS and fixed with 1:1 4% paraformaldehyde in 2% FCS/PBS. eMSC were analyzed using BD FACSCanto II (BD) (10,000 events/sample) and FlowJo v.10 software.

### Colony-Forming Unit-Fibroblast (CFU-F) Assay

eMSC were cultured with or without A83-01 for 3 passages (36 days) and then seeded at 50 cells/cm^2^ to assess cloning efficiency as described previously (Gurung et al., 2015), with minor modifications. Briefly, eMSC were seeded on fibronectin-coated 10 mm culture dishes and cultured in SFM, supplemented with bFGF and EGF (both 10 ng/mL; Invitrogen), in the presence or absence of A83-01 (1 μM) in SFM at 37°C in 5% CO_2_, 5% O_2_, 90% N_2_ for 2 weeks, with weekly medium changes. Cultures were then fixed in 10% formalin and stained with hematoxylin (Amber Scientific, United States), washed and blued in Scott’s tap water. Colonies were counted and colony efficiency determined by dividing total number of colonies by number of cells seeded and multiplied by 100.

### RNA Extraction, RNA Quality Control and RNA Libraries

Total RNA was extracted from untreated and A83-01-treated eMSC using RNeasy Mini kit (Qiagen, Germany) according to the manufacturer’s instructions, with some variations. Briefly, eMSC were trypsinised using TrypLE^TM^ (Life Technologies), resuspended in DMEM/F12 containing 5% Albumax II and then centrifuged at 300 *g* for 5 min. Cell were lysed with RNeasy Lysis Buffer, genomic DNA contamination removed with RNase-free DNase (Qiagen) and RNA eluted with 30–50 μl RNase-free water. RNA quality was assessed on an Agilent Technologies 2100 Bioanalyzer according to the manufacturer’s instructions. Samples with RNA Integrity Number (RIN) > 8 were subjected TruSeq Poly-A mRNA Library Pro Kit protocol 15031047 RevD (Illumina, United States) to generate indexed cDNA libraries. The library size was assessed on an Agilent Bioanalyzer and quantified by Qubit and qPCR.

### RNA-Seq

RNA libraries were sequenced by Illumina HiSeq3000. Fifty million single-end reads were sequenced per sample with a read length of 50 bp. Transcriptomic maps were identified using Bowtie-2.2.3 (Langmead and Salzberg, 2012) and Samtools-1.2.0 (Li et al., 2009) against the UCSC hg19 transcriptome reference from the Illumina iGenomes resource. Counts were assessed using HTSeq -0.6.1 (Anders et al., 2015) and transcripts per million (TPM) were calculated. DESeq2 (Anders and Huber, 2010) was used for detection of differentially expressed genes in a pair-wise manner. Differentially expressed genes were subjected to Gene Ontology (GO) and KEGG Pathway enrichment analyses using the Database for Annotation, Visualization and Integrated Discovery (DAVID) version 6.8 (Huang da et al., 2009) and visualized using Reduce and Visualize Gene Ontology (REVIGO) online software (Supek et al., 2011). Fastq, metadata spreadsheet and table of counts have been deposited in the National Centre for Biotechnology Information Gene Expression Omnibus/sequence Read Archive with GEO (NCBI) accession number SuperSeries GSE146067/SubSeries GSE146066.

### ATAC-Seq

ATAC-seq was performed as described (Buenrostro et al., 2013), although with some modifications (Vrljicak et al., 2018). Briefly, cells were washed with cold PBS, lysed using cold EZ lysis buffer (10 mM Tris-HCl, pH 7.4, 10 mM NaCl, 3 mM MgCl2 and 0.1% IGEPAL CA-630, Sigma-Aldrich, United Kingdom), transferred to chilled nuclease-free tubes, vortexed, left on ice for 5 min, and then pelleted in a refrigerated centrifuge. The nuclear pellet was washed in EZ lysis buffer and re-suspended in the transposase reaction mix containing 25 μl Tagment DNA (TD) Buffer, 5 μl Tagment DNA Enzyme and 20 μl nuclease free water (Nextera DNA Sample Preparation Kit, Illumina, United Kingdom) for 45 min at 37°C. Samples were purified using a Zymo DNA Clean and Concentrator-5 Purification kit (Zymo Research, United States). Briefly, DNA binding buffer was added to 50 μl samples, mixed and transferred to the column, centrifuged at 17,000 *g* for 30 s at RT, 200 μl DNA wash buffer added, columns centrifuged and repeated twice. After removing the residual liquid, 23 μl pre-warmed elution buffer was added and incubated for 2 min at RT, then centrifuged for 2 min to elute DNA. Twenty μl samples were added to PCR tubes containing 5 μl index 1, 5 μl index 2, 15 μl Master mix (NPM), 5 μl Primer Cocktail (Nextera DNA Sample Preparation Kit and Nextera Index Kit, Illumina). Amplification was performed in a Veriti 96 Well Thermal Cycler (Applied Biosystems, United States) using the following PCR conditions: 72°C for 3 min, 98°C for 30 s then 15 cycles of 98°C for 10 s, 63°C for 30 s, 72°C for 1 min. Libraries were purified using AMPure XP beads using the Illumina Nextera kit recommended protocol and quantified using Qubit HS DNA Assay on a Qubit 2.0 Fluorometer. Library sizes were assessed by Agilent Bioanalyzer using the High Sensitivity DNA chip. ATAC-seq library samples were sequenced on an Illumina HiSeq 1500 to a depth of thirty million paired-end reads/sample, with a read length of 100 bp. ATACseq data from this study have been deposited in the GEO (NCBI) under the accession number SuperSeries GSE146067/SubSeries146065.

### ATAC-Seq Data and Motif Analyses

Sequenced paired-end reads were aligned to the University of California Santa Cruz (UCSC) human genome 19 (hg19) assembly using Bowtie2-2.2.6 (Langmead and Salzberg, 2012) and Samtools-1.2.0 (Li et al., 2009) and peak calling performed using MACS-2.1.0. HTSeq-0.6.1 (Anders et al., 2015) to count the reads overlapping the peaks and differential expression analysis of sequencing data 2 (DESeq2) was used to determine opening and closing regions of the chromatin (Anders and Huber, 2010). Fastq, metadata spreadsheet and table of counts have been deposited in the National Centre for Biotechnology Information Gene Expression Omnibus/sequence Read Archive with GEO accession number GSE146065. Differential open chromatin regions were mapped to *cis*-regulatory elements of their proximal genes using ENCODE DNaseI hypersensitivity data (Thurman et al., 2012). Physical interaction and distance no greater than 10 kb were used as criteria to assess association between ATAC-seq peak and proximal gene regulatory element. *De novo* short sequence motif analysis using Hypergeometric Optimization of Motif Enrichment (HOMER) v.4.8 was performed on 3,555 opening and 2,412 closing ATAC-seq peaks to determine enrichment and depletion of TF short sequence binding motifs in the differential ATAC-seq peaks (Heinz et al., 2010).

### RARβ Inhibition Experiments

Passage 3 cultured eMSC from 4 biological samples were treated with 1 μM A83-01 or 0.01% DMSO (vehicle control) for 7 days, then trypsinized and seeded in triplicate at 1000 cells/well (3.125 × 10^3^ cells/cm^2^) into fibronectin-coated wells of two 96 well plates per biological replicate to assess proliferation on day 0 (D0) and day 3 (D3) in the presence or absence of 10 μM RARβ antagonist LE135 (Tocris Bioscience) using the cell viability MTS assay. After seeding, cells were allowed to adhere for 1 h before addition of 100 μl medium containing A83-01 + LE135 or A83-01 + DMSO. After 2 h, the MTS reagent (20 μl, CellTiter 96 AQueous One Solution Cell Proliferation Assay, Promega, United States) was added to wells for the D0 timepoint and incubated for 2.25 h in the dark, then absorbance read at 490 nm on a spectrophotometer (Spectramax i3, Molecular Devices, United States). The medium was changed on the D3 plate at 24 h and the viability assay completed 72 h after seeding, as described above. Parallel cultures were subjected to CFU-F assays. Data were corrected for background readings (medium only) for each plate and normalized to D0 A83-01 for each sample and reported as fold-change.

### Statistical Analysis

Statistical analyses were performed with GraphPad Prism 8. Technical replicates were inspected for outliers, which were removed from the analysis using Grubbs test. Normality of the data was determined with Shapiro–Wilk normality test. Individual data points and mean ± standard error of the mean (SEM) are shown when appropriate. Statistical significance was determined using two-way analysis of variance (ANOVA) or ratio paired *t*-tests with *p* < 0.05 considered statistically significant. For RNA-seq data analysis, statistical significance was assessed using the Benjamini–Hochberg procedure to control false discovery rate. Changes in gene expression were deemed statistically significant if the adjusted *p*-value (*q*-value) was less than 0.05.

## Results

### Phenotypic Characterization of Sustained A83-01-Treated eMSC

**FIGURE 1:**
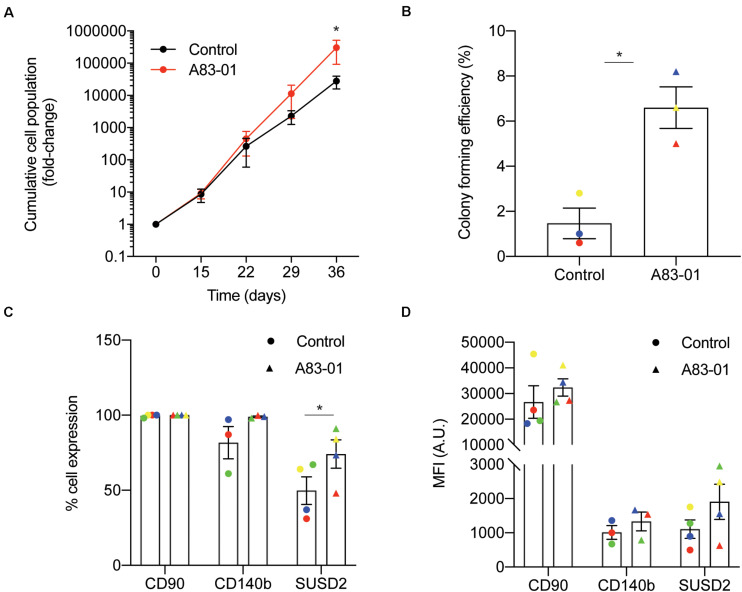
Sustained TGFβ-R inhibition modulates eMSC function and phenotype in culture. Primary human eMSC, isolated using SUSD2 magnetic bead sorting, were cultured with or without 1 μM A83-01 in 5% O2 for 36 days. **(A)** Cumulative cell population in eMSC cultures. Data are mean ± SEM; *p < 0.05. Note the logarithmic scale of the *Y*-axis. **(B)** Clonogenicity of 3 independent MSC cultures following 36 days culture in SFM with and without A83-01. Surface phenotype assessed by flow cytometry of eMSC cultures after 36 days with or without A83-01 shown as **(C)** % positive cells and **(D)** MFI for CD90, CD140b and SUSD2. Data are mean ± SEM of *n* = 4 (except for CD140b where *n* = 3) individual eMSC lines. Individual samples are shown in as different colored data points. *p = 0.039 from control.

We previously demonstrated that late passage eMSC cultured for just 7 days with the TGFβ-R signaling pathway inhibitor, A83-01, in SFM retain their SUSD2^+^ phenotype and function (Gurung et al., 2015). Apoptosis and senescence were prevented in these short term A83-01-cultured eMSC. To investigate if eMSC can be expanded more efficiently when maintained under sustained TGFβ-R inhibition from culture initiation, cells isolated from 3 individual biopsies were seeded at 5000 cells/cm^2^ in SFM supplemented with either A83-01 or vehicle (DMSO). The medium was refreshed every 48 h and cumulative cell population calculated at each passage (culture days 15, 22, 29, and 36). As shown in Figure 1A, treatment of eMSC with A83-01 from culture initiation progressively conferred a proliferative advantage. After 36 days in culture, at least one order of magnitude more cells were produced in the A83-01 medium when compared to control medium (Figure 1A). Next, we performed colony-forming unit-fibroblast (CFU-F) assays on eMSC first cultured with or without A83-01 for 3 passages (36 days) and then seeded at a low density (50 cells/cm2) to allow colony formation for a further 14 days. In keeping with our previous study (Gurung et al., 2015), exposure of cultured eMSC to A83-01 increased the CFU-F activity of primary cultures between ∼ 4-10-fold (*p* = 0.0146; Figure 1B). We then chose the 36-day timepoint (Figure 1A) to analyze A83-01-treated cells for the expression of phenotypic eMSC markers CD140b, SUSD2, and CD90 as a representative ISCT marker, by flow cytometry. The abundance of CD90^+^ cells was 99.1 ± 0.5% (*n* = 4) for the control and did not change upon TGFβ-R blockade (99.7 ± 0.2%, *n* = 4) (Figure 1C). Similarly, CD140b did not change following prolonged A83-01 treatment (81.7 ± 10.7% vs. 98.9 ± 0.5% (*n* = 3). In contrast, A83-01 treatment increased the abundance of SUSD2^+^ cells from 49.8 ± 9.2% to 74.1 ± 9.4% (*n* = 4, *p* = 0.011) (Figure 1C). Mean fluorescence intensity (MFI) was also calculated to evaluate the abundance of different cell surface molecules/cell. The MFI for CD90, CD140b and SUSD2 did not increase (Figure 1D) in response to prolonged A83-01 treatment, although there was a trend for SUSD2 (1108 ± 269 vs. 1907 ± 516, *n* = 4, *p* = 0.089).

### Transcriptional Profiling of Sustained A83-01-Treated eMSC

**FIGURE 2:**
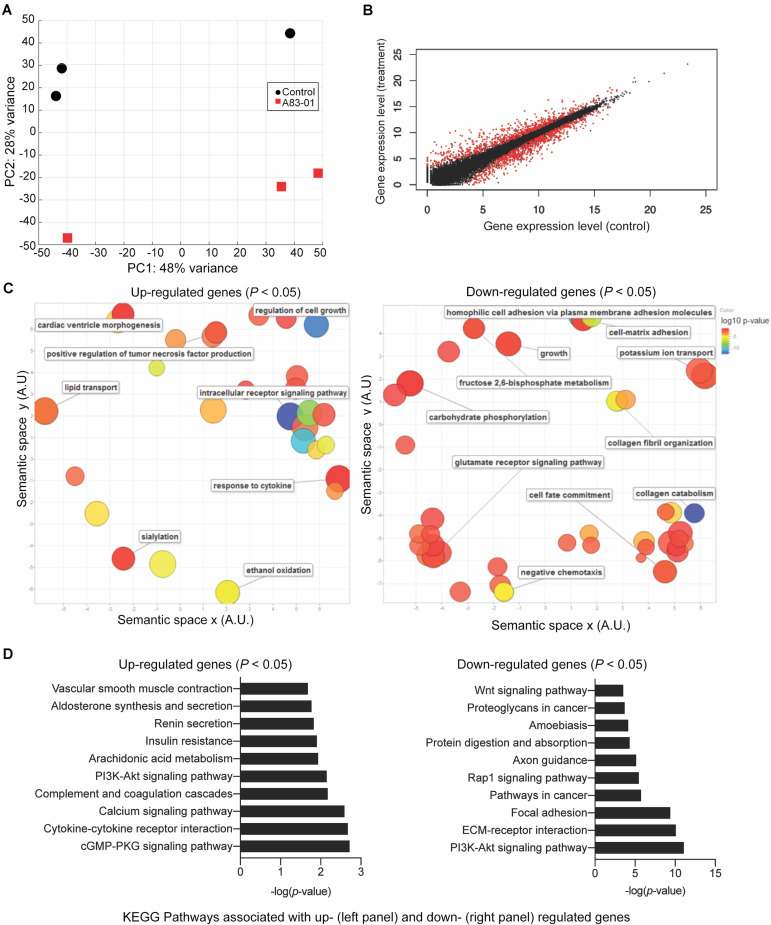
Transcriptomic profile of eMSC upon sustained A83-01 treatment. **(A)** Principal component analysis of RNA-seq data from three independent primary eMSC cultures treated with or without TGFβ-R inhibitor for 36 days. **(B)** Average gene expression levels expressed as log2 transformed counts normalized to library size between control and A83-01 treated libraries. Red dots represent significantly differentially expressed genes (*q* < 0.05), black dots indicate non-differentially expressed genes. **(C)** Semantic clustering of significantly overrepresented GO terms (*p* < 0.05) of differentially induced and repressed genes (left and right panel, respectively) upon A83-01 treatment. The color key is shown on the right. The most highly enriched GO categories are indicated in blue. The size of the circles reflects the frequency of the GO term. **(D)** Enrichment analysis of KEGG pathways associated with up- and down-regulated genes (left and right panels, respectively).

To explore how cultured eMSC are maintained in a more naïve state upon sustained TGFβ-R inhibition, three independent cultures treated with or without A83-01 for 36 days were subjected to RNA-seq. Principal component analysis revealed that the greatest variation in gene expression is accounted for by intrinsic differences between primary cultures. The effect of A83-01 treatment was apparent in principal component 2 (PC2), which accounted for 28% of the variance in gene expression (Figure 2A). Following Benjamini–Hochberg correction for multiple testing, 1,463 genes were differentially expressed upon A83-01 treatment (Figure 2B), 759 (52%) of which were up-regulated and 704 (48%) down-regulated. Gene ontology (GO) analysis using DAVID revealed that genes induced upon A83-01 treatment are enriched in 61 biological processes, including ‘regulation of cell growth’ (*p* = 7.8 × 10^–4^) and ‘intracellular receptor signaling pathways’ (*p* = 4.5 × 10^–4^) (Figure 2C, left panel). Conversely, analysis of downregulated genes yielded GO terms such as ‘cell fate commitment’ (*p* = 4.9 × 10^–4^), ‘collagen catabolism’ (*p* = 1.7 × 10^–13^), and ‘collagen fibril organization’ (*p* = 4.6 × 10^–6^) (Figure 2C, right panel). Notably, 20 out of the 43 most significantly down-regulated genes in A83-01 treated eMSC encode extracellular matrix (ECM) components, including various collagen subunits (e.g., *COL1A1*, *COL1A2*, *COL4A1*, *COL4A2*, *COL5A1*, *COL5A2*, *COL6A3*, and *COL8A1*), secreted protein acidic and cysteine rich (*SPARC*), and fibronectin (*FN*) (Supplementary Figure S1). Many of the ECM genes repressed by A83-01 are very highly expressed in untreated eMSC with levels ranging from 342 to 14,586 TPM (Supplementary Figure S1). These data suggest that the effect of TGFβ-R blockade on eMSC is mediated, at least in part, by limiting ECM synthesis and deposition in prolonged culture. As shown in Supplementary Figure S2, several angiogenic, anti-inflammatory, immunomodulatory, antifibrotic and anti-apoptotic genes were significantly upregulated in A83-01-treated cells, in keeping with our previous report (Gurung et al., 2018b).

Further annotation of differentially expressed genes using the KEGG Pathway database underscored the functional differences between A83-01-treated and untreated cultures (Figure 2D). Notable pathways enriched in eMSC in response to sustained TGFβ-R inhibition included the ‘cyclic guanosine monophosphate (cGMP)-protein kinase G (PKG) signaling pathway,’ which is implicated in nitric oxide-mediated cardioprotection during acute ischemic preconditioning (Sun et al., 2013), cell growth, and inhibition of apoptosis (Wolfertstetter et al., 2013), and ‘cytokine-cytokine receptor interaction’, in keeping with the innate paracrine function of MSC (Tolar et al., 2010; Mukherjee et al., 2019a). Conversely, A83-01 repressed genes were enriched in ‘pathways in cancer,’ ‘focal adhesion,’ and ‘ECM-receptor interaction.’ Notably, the ‘PI3K-Akt signaling pathway’ was common to both up- and down-regulated genes. This multifaceted pathway controls key cellular processes by phosphorylating substrates involved in apoptosis, protein synthesis, metabolism, and cell cycle. Mining of the RNA-seq data revealed that A83-01 induces several genes involved in activation of response to oxidative stress and cellular detoxification pathways, including Prostaglandin-Endoperoxide Synthase 2 (*PTGS2*), Cytoglobin (*CYGB*), Scavenger Receptor Class A Member 3 (*SCARA3*), Glutathione Peroxidase 3 (*GPX3)*, Scavenger Receptor Class A Member 5 *(SCARA5)* and Apolipoprotein E (*APOE*). Conversely, multiple senescence-associated genes, including Cyclin Dependent Kinase Inhibitor 1A (CDKN1A), Cyclin Dependent Kinase Inhibitor 2B (*CDKN2B*), Cyclin Dependent Kinase 6 (*CDK6*), Cyclin D2 (*CCND2*), Ataxia Telangiectasia Mutated (ATM), tumor protein p53 inducible protein 3 (*TP53I3*) and phosphatase and tensin homolog (*PTEN*) were inhibited upon A83-01treatment (Supplementary Table S1 and Supplementary Figures S3A,B). Other senescence associated genes, such as Serpin Family E Member 2 *(SERPINE2)*, and Netrin 4 *(NTN4)* (Supplementary Figures S3A,B) were also significantly downregulated. This gene signature supports the conjecture that continuous TGFβ-R inhibition during culture expansion leads to the emergence of senescence-resistant eMSC.

### Chromatin Changes Induced by Sustained A83-01 Treatment of eMSC

**FIGURE 3:**
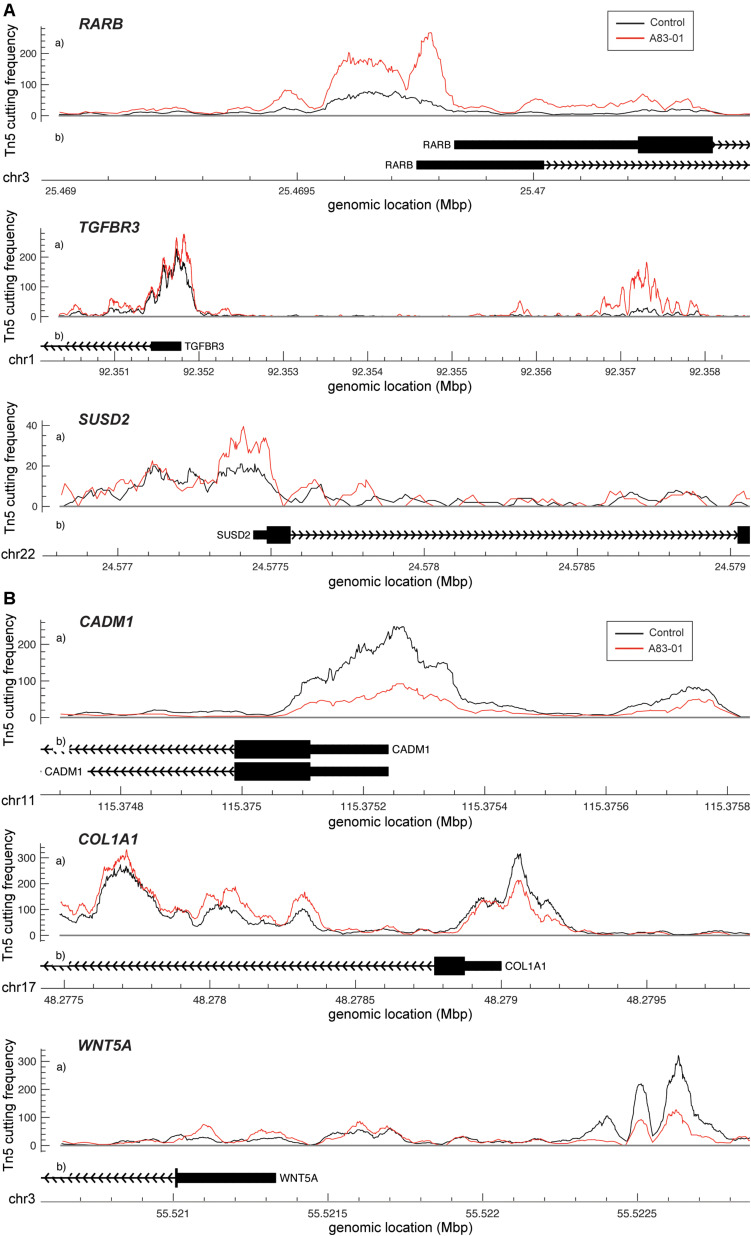
Changes in chromatin accessibility in A83-01 treated eMSC. **(A)** Representative ATAC-seq peaks showing transition from closing to opening chromatin upstream of the promoter of RARB, TGFBR3 and SUSD2 in response to A83-01. **(B)** Representative ATAC-seq peaks showing transition from opening to closing chromatin of the proximal promoter of CADM1 and COL1A, and of a distal enhancer of WNT5A in response to A83-01. Black and red traces represent untreated and A83-01-treated eMSC cultures. The *X*-axis shows the genomic location of the ATAC-seq peaks and genes. The *Y*-axis shows the frequency of Tn5 cutting.

Dynamic changes in chromatin structure and epigenetic code drive gene expression and ultimately define cell identity (Chen and Dent, 2014). To map the global changes in the genomic architecture of cultured eMSC in response to sustained TGFβ-R inhibition, 3 independent eMSC cultures treated with or without A83-01 for 36 days were subjected to ATAC-seq, which profiles chromatin accessible regions as a sequencing depth readout (Vrljicak et al., 2018). Based on *q* ≤ 0.05, DESeq identified 5,967 differential ATAC-seq peaks upon A83-01 treatment, 60% of which involved opening of genomic regions and 40% closing of specific loci. Out of 5,967 peaks, 31 and 29% of the opening and closing ATAC-seq peaks, respectively, fell within −10 to +1 kilobases (kb) around transcriptional start sites (TSSs). *RARB* (coding retinoic acid receptor beta, RARβ), *TGFBR3* (transforming growth factor beta receptor 3) and *SUSD2* exemplify genes that showed increased chromatin accessibility at and upstream of their proximal promoters upon A83-01 treatment (Figure 3A). Cross-referencing with RNA-seq data showed a significant increase in *RARB*, *TGFBR3* and *SUSD2* transcript levels in response to A83-01 treatment (*q* = 1.1 × 10^–33^, *q* = 1.2 × 10^–22^, and *q* = 2.7 × 10^–5^, respectively). Conversely, *CADM1* (cell adhesion molecule 1), *COL1A1* (collagen type I alpha 1 chain), and *WNT5A* are examples of genes repressed in response to A83-01 treatment (*q* = 1.9 × 10^–46^, *q* = 1.4 × 10^–9^, and *q* = 5.0 × 10^–30^, respectively). As shown in Figure 3B, downregulation of *CADM1* and *COL1A1* is associated with closure of their proximal promoters whereas silencing of *WNT5A* coincides with closure of a distal enhancer. Given that A83-01 inhibits senescence and apoptosis upon expansion of eMSC in culture (Gurung et al., 2015), we also searched for differential ATAC-seq peaks in proximal promoters of genes involved in senescence resistance. For example, A83-01 treatment resulted in opening of the proximal *PGTS2* (prostaglandin endoperoxidase synthase 2) promoter (*q* = 5.4 × 10^–2^). On the other hand, loss of chromatin accessibility to the *CCND2* (cyclin D2) promoter (*q* = 1.1 × 10^–2^) (Supplementary Figure S3C) was also observed.

**FIGURE 4:**
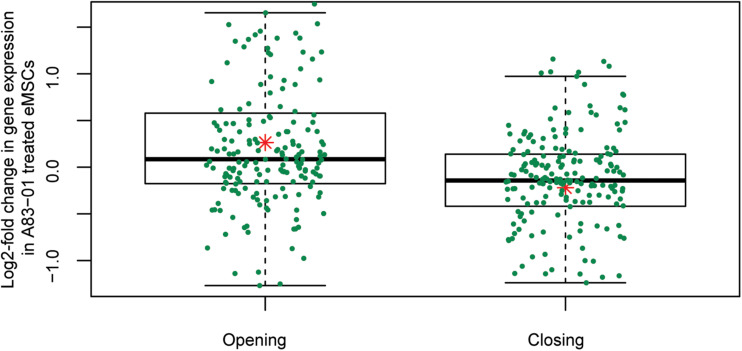
Differential chromatin opening correlates with gene expression changes in A83-01-treated eMSC. Changes in chromatin landscape of eMSC in response to TGFβ-R inhibition correlate with differential regulation of gene expression. Box plots showing increase or decrease in transcript levels of 200 genes (within 10 kb of the TSS) associated with the most open and closed ATAC-seq peaks. *Y*-axis shows relative changes in transcript levels, expressed as log2-fold change: +ve and –ve values relate to up- and down-regulated genes, respectively. *X*-axis shows ATAC-seq peaks clustered in opening and closing peaks. Green dots represent the genes and the red asterisk represents mean log2-fold change (*p* = 1.0 × 10-6, *t*-test).

The gain or loss of ATAC-seq peaks upon A83-01 treatment indicates that altered transcription factor (TF) binding drives differential gene expression. However, both activating and repressive TFs can potentially bind at different regulatory sites, rendering it challenging to confidently predict gene expression from dynamic chromatin changes at specific loci alone (Vrljicak et al., 2018). Nevertheless, analysis of 200 genes associated with the most induced or repressed ATAC-seq peaks (within 10 kb of TSSs) revealed a strong association with increased or decreased expression, respectively, upon A83-01 treatment (*p* = 1.0 × 10^–6^) (Figure 4). Next, we interrogated the ATAC-seq data to gain insight into the *cis*- regulatory landscape that underpins the transcriptional responses of eMSC to sustained A83-01 treatment from culture initiation. *De novo* binding motif enrichment analysis was performed using HOMER on 3,555 opening and 2,412 closing ATAC-seq peaks. This annotation yielded 19 significantly overrepresented motifs in opening peaks (Supplementary Figure S4), and 17 overrepresented motifs in closing peaks (Supplementary Figure S5).

**FIGURE 5:**
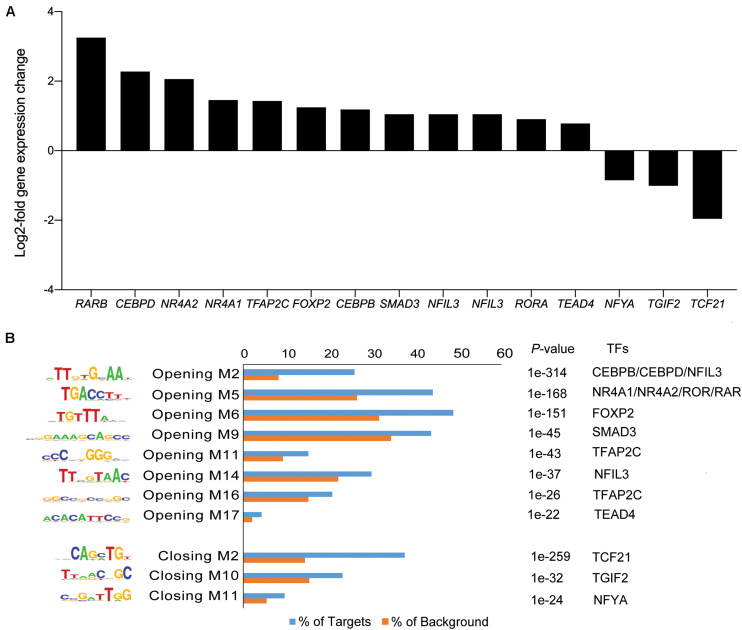
Motif discovery analysis in dynamic genomic regions. **(A)** Inhibition of TGFβ-R signaling pathway alters expression of genes encoding TFs. Graph shows expression of selected significantly up- and down-regulated TFs (log2-fold change ≥ 1 and ≤ -1). **(B)** Differentially regulated TFs matched to enriched and depleted short sequence binding motifs. Bar graph showing enriched and depleted binding motifs coupled with the most plausible differentially expressed TFs, based on motif specificity. In the bar graph, the frequency (%) of peaks (blue bars) containing the motif is shown relative to genomic regions randomly selected from the genome (orange bars) (±50 kb from TSS, matching size, and GC/CpG content). P indicates the *p*-value of the short sequence binding motifs.

Next, we matched the motifs against canonical TFs that are differentially expressed in cultured eMSC treated with and without A83-01 (Figure 5A). CCAAT/enhancer binding protein beta and delta (*CEBPB*/*CEBPD*), *RARB*, RAR-related orphan receptor alpha (*RORA*), and nuclear receptor subfamily 4 group A member 1 (*NR4A1*, also known as *NUR77*) were amongst the most plausible differentially expressed genes of TFs that can bind the enriched motifs in opening ATAC-seq peaks with high affinity (Figures 5A,B). Conversely, reduced expression of transcription factor 21 (*TCF21*), TGFB induced factor homeobox 2 (*TGIF2*), and nuclear transcription factor Y subunit alpha (*NFYA*) paralleled the loss of their corresponding binding sites in closing loci (Figures 5A,B).

### RARβ Is One Mediator of A83-01 Responses in eMSC

**FIGURE 6:**
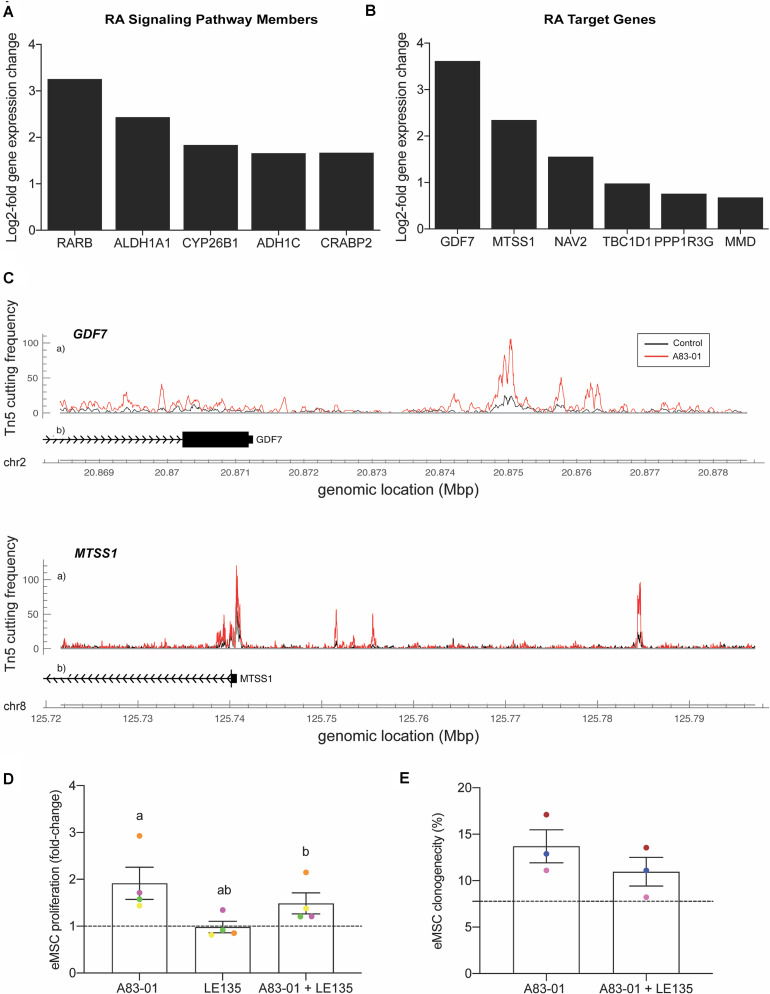
RARβ is one mediator of A83-01 responses in eMSC. **(A)** A83-01 treatment induces genes implicated in RA signaling and **(B)** RA downstream target genes. **(C)** Examples of RA target genes with increased chromatin accessibility. **(D)** Inhibition of RARβ using a selective antagonist, LE135, partially reverses the proliferation advantage gained upon A83-01 treatment and **(E)** reduces colony forming efficiency. Data in **(D,E)** are mean ± SEM of fold change over control (shown as dotted line). Individual samples are shown in as different colored data points. Shared symbols indicate significant differences, *p* < 0.05.

A notable observation was that A83-01 treatment markedly upregulated *RARB* expression (*q* = 1.1 × 10^–33^) (Figure 5A) in parallel with genome-wide enrichment of RAR/RORA binding sites (*p* = 1.0 × 10^–168^) (Figure 5B). RARβ is a member of the thyroid-steroid hormone receptor superfamily of nuclear transcription regulators. It binds retinoic acid (RA), the biologically active form of vitamin A (Gudas, 2012). Further, A83-01 induces multiple other genes implicated in RA signaling (Figure 6A), including the cellular retinoic acid binding protein 2 (*CRABP2*), as well as known RA target genes (Figure 6B). *GDF7* (coding growth differentiation factor 7) and *MTSS1* (MTSS I-BAR domain containing 1) exemplify RA target genes that exhibited increased chromatin accessibility and expression in A83-01-treated cells (Figure 6C). To explore the effect of RARB on A83-01-treated eMSC further, we utilized LE135, a selective RARβ antagonist (Murakami et al., 2013). LE135 had no effect on eMSC proliferation in standard cultures not treated with A83-01. However, addition of LE135 to A83-01 treated cultures indicated that RARβ inhibition partially reverses the proliferation advantage conferred by TGFβ-R inhibition (Figure 6D). In addition, LE135 reduced the clonogenicity of A83-01 treated eMSC (Figure 6E).

## Discussion

This study demonstrates that eMSC cultured under continuous TGFβ-R inhibition from culture initiation are protected against loss of proliferation and clonogenicity upon long-term expansion. The impact of A83-01 on the phenotype of cultured eMSC was underpinned by an altered chromatin landscape and gene expression. More than 1,400 genes were differentially regulated by culturing eMSC in A83-01-containing SFM for 5 weeks, characterized by upregulation of angiogenic, anti-inflammatory, immunomodulatory, antifibrotic and antiapoptotic genes and marked downregulation of ECM genes. The overall signature indicated that A83-01 maintains the expression of genes involved in eMSC paracrine activity while simultaneously inhibiting activation of fibroblast differentiation genes. Over 3,500 regions of chromatin opened while approximately 2,400 genomic loci closed in response to continuous TGFβ-R inhibition. Motif analysis identified marked enrichment of several putative TF binding sites associated with the more undifferentiated A83-01-treated eMSC, most prominently RAR/ROR. *RARB* expression was also markedly upregulated in parallel with multiple genes encoding signal intermediates in the RA pathway as well as known RA target genes. Functional assays with a selective RARB inhibitor demonstrated for the first time an integral role for RA signaling in effecting eMSC responses to prolonged A83-01 treatment. Thus, sustained inhibition of TGFβ-R signaling during eMSC expansion in extended serum-free cultures under physiological O_2_ conditions produces a relatively homogeneous, undifferentiated population of cells with favorable properties for clinical translation.

We reported previously that short-term treatment with A83-01 in SFM (7 days) following extensive culture expansion of eMSC in serum medium (6 passages) increases proliferation and enhances clonogenicity (Gurung et al., 2015). Here we report that sustained TGFβ-R inhibition from culture initiation similarly promotes eMSC clonogenicity and proliferation with significant differences observed by passage 3. Although eMSC intrinsically have extensive proliferative capacity in culture, the use of A83-01 enhances this proliferative capacity, increasing cellular output for potential clinical translation (Gargett et al., 2019). Prolonged culture under these conditions also increased the percentage of SUSD2-expressing cells, but not CD140b or the representative ISCT marker CD90, nor the density of these surface markers on the A83-01-treated cells, indicating that the perivascular eMSC phenotype was retained and spontaneous fibroblast differentiation attenuated.

We reported previously that the MFI and percentage of cells positive for certain ISCT surface markers (CD29, CD44, CD73, CD105) are insensitive to culture conditions (Rajaraman et al., 2013). This is also the case for CD90. Similar observations have been reported for adipose, dental pulp, amniotic fluid MSC (Gharibi and Hughes, 2012) and bone marrow MSC (Haniffa et al., 2009). Further, ISCT markers do not discriminate between MSC and differentiated fibroblasts in culture (Haniffa et al., 2009). Likewise, CD90 is expressed *in vivo* on human endometrial fibroblasts as well as eMSC (Schwab et al., 2008), supporting our *in vitro* observations. By contrast, we have provided evidence that more recent bone marrow MSC markers, such as *TWIST1*, *TWIST2*, and *JAG1* (Gurung et al., 2018b), are more indicative of eMSC function. Notably, these marker genes are upregulated within 1 week of culturing in A83-01-containing medium (Gurung et al., 2018b). Currently, SUSD2 is the most sensitive marker to distinguish eMSC from endometrial fibroblasts with the level of expression correlating to the abundance of highly proliferative and clonogenic eMSC in culture (Murakami et al., 2014; Gurung et al., 2015; Bozorgmehr et al., 2020).

Our data for eMSC clonogenicity and proliferation also highlighted intrinsic differences in A83-01 responsiveness between eMSC cultures established from different donors, a well-known feature of primary MSC cultures (Gargett et al., 2016). Recently it was shown that body mass index of patients, but not age, correlate inversely with the abundance of clonal SUSD2^+^ eMSC (Murakami et al., 2013). These observations raise the possibility that clinical variables, such as obesity, impact on the *in vitro* expandability of eMSC.

The divergent expression of a large number of genes in A83-01-treated cultures suggests either profound transcriptional reprogramming or – perhaps more likely – attenuation of fibroblast differentiation cues imposed by *in vitro* culture conditions. For example, a striking but not entirely unexpected observation is that A83-01 represses multiple highly expressed genes involved in ECM deposition and collagen metabolism, including 8 collagen subunit genes, *FN1*, *TGFB1* and *SPARC*. During normal wound healing, TGFβ signaling is transiently increased to activate fibroblasts (Massague, 2012). Failure to terminate TGFβ signaling following tissue repair results in chronic activation of fibroblasts, massive accumulation of ECM, and fibrosis. Thus, a likely mechanism of A83-01 actions involves prevention of illicit fibroblast differentiation and activation in extended eMSC cultures. In keeping with previous observations (Gurung et al., 2018b), genes involved in angiogenesis (e.g., *SLIT2*, *HPSE*, and *SFRP1*) (Dufourcq et al., 2008; Urbich et al., 2009; Lv et al., 2018), cell survival (e.g., *ENPP2*, *FAIM2*) (Somia et al., 1999; Vidot et al., 2010), and immunomodulation (e.g., *TLR3* and *IL1R1*) (Rameshwar et al., 1997; Farkas et al., 2019) were also upregulated upon prolonged A83-01 treatment. This gene profile infers that sustained TGFβ-R inhibition leads to expansion of perivascular eMSC that are more efficient in reducing inflammation, promoting wound healing and tissue growth, and minimizing fibrosis when transplanted *in vivo*. These functional properties are considered cardinal features of MSC, which were recently renamed as Medicinal Signaling Cells to reflect their paracrine activity (Caplan, 2017).

ATAC-seq analysis revealed that TGFβ-R inhibition in cultured eMSC modifies chromatin accessibility at almost 6,000 genomic regions. The overall pattern, characterized by more opening than closing loci, is in keeping with the less differentiated state of A83-01 treated cells (Chen and Dent, 2014). A strong correlation was observed between changes in gene expression and differentially chromatin accessibility of promoter regions. For example, opening of chromatin at and upstream of the SUSD2 promoter corresponded to increased abundance of SUSD2^+^ eMSC in A83-01 cultures. Interestingly, SUSD2 has been shown to prevent senescence and cell death in tumor cells (Zhang et al., 2017), suggesting its induction is important for expansion of cultured eMSC.

Furthermore, maintenance of eMSC lifespan during culture expansion under sustained TGFβR inhibition involves upregulation of antioxidant pathways and downregulation of cell cycle arrest pathways that lead to the senescent phenotype of aging MSC (Martínez-Zamudio et al., 2017; Wiese et al., 2019; Cakouros and Gronthos, 2020). For example, A83-01 treatment resulted in opening of the proximal *PGTS2* promoter in parallel with increased transcription. By contrast, expression of multiple transcripts involved in the cyclin-dependent kinase inhibitor pathway were repressed, including *CCND2*, which also showed proximal promoter closing and is associated with MSC senescence (Bertolo et al., 2019). A83-01 effects on chromatin architecture and gene expression may also prevent transcriptome drift that precedes MSC aging (Wiese et al., 2019).

Another potential mechanism that promotes survival of A83-01-treated eMSC involves closure of the proximal promoter of *CADM1*, which encodes a potent inhibitor of cell proliferation and migration (Murakami, 2005). *CADM1* repression is mediated by TWIST1 (Hartsough et al., 2019), a TF upregulated in eMSC in response to A83-01 treatment (Gurung et al., 2018b). The altered cis-regulatory chromatin landscape in response to A83-01-induced TGFβ-R blockade also suggested that silencing of specific TFs may be essential to maintain cultured eMSC in an undifferentiated state. A case in point is the loss of TCF21 binding sites in parallel with marked repression of *TCF21* expression. TCF21 is a member of basic helix-loop-helix family of transcription factors, which orchestrates cell-fate specification, commitment and differentiation in multiple cell lineages during development (Acharya et al., 2012). Furthermore, TCF21 has recently been shown to drive fibrosis associated with ovarian and deep infiltrating endometriosis (Ganieva et al., 2020).

Our combined RNA- and ATAC-seq analysis identified the RA pathway as a putative pharmacological target to modulate eMSC in culture. A83-01 markedly upregulated *RARB* expression in parallel with genome-wide enrichment of putative RORA/RAR binding sites. RA plays an important role during embryonic and fetal development and fine-tunes cellular immune response (Mora et al., 2008). In human endometrium, RA signaling is silenced upon differentiation of endometrial fibroblasts into specialized decidual cells (Vrljicak et al., 2018). Induction of RA target genes in A83-01-treated eMSC confirmed that this signaling pathway is activated endogenously in response to sustained TGFβ-R inhibition. Further, the selective RARβ antagonist, LE135, attenuated the cellular responses to A83-01, indicating that increased or sustained RA-RARβ signaling protects eMSC against loss of proliferative capacity and clonogenicity in prolonged culture. Another putative pharmacological target is NR4A1, an orphan nuclear receptor highly induced in A83-01 treated cells. This orphan nuclear receptor exerts pleiotropic regulatory effects on glucose and lipid metabolism (Pei et al., 2006), inflammatory responses (Fassett et al., 2012), and vascular homeostasis (Zeng et al., 2006). Interestingly, NR4A1 inhibits TGFβ signaling in the nucleus by promoting the assembly of a repressor complex that binds to the promoters of TGFβ target genes (Palumbo-Zerr et al., 2015). Because of its role as an endogenous TGFβ inhibitor, small-molecule NR4A1 agonists are under development for the treatment of fibrotic disorders (Palumbo-Zerr et al., 2015).

While the present study shows that A83-01-pretreated eMSC show promise for clinical translation, further research is required to determine their quality, safety and efficacy profile. This includes determining the minimal release criteria such as %SUSD2^+^ cells, secreted proteins and qRT-PCR of key genes that predict efficacy of *in vivo* function of A83-01-pretreated eMSC. We will also assess the effect of A83-01 withdrawal and reversibility on global chromatin architectural changes and gene expression, and the effect on cryopreservation and thawing. Functional *in vivo* assays of sustained A83-01-treated eMSC will be assessed using our xenograft mouse model (Gurung et al., 2018a) and in tumorigenicity animal models (Sato et al., 2019). *In vitro* potency assays that reflect *in vivo* eMSC function need to be developed, based on their angiogenic and immunomodulatory properties.

In summary, by integrating genome-wide expression and DNA accessibility profiling techniques, this study has advanced our understanding of the *cis*-regulatory DNA landscape and gene networks that safeguards eMSC against spontaneous fibroblast differentiation and loss of function in prolonged cultures. Analyses of these two large data sets revealed novel pharmacological targets that could be exploited to accelerate clinical translation of autologous eMSC therapies for a variety of reproductive disorders. Furthermore, the data sets constitute a robust resource to interrogate fundamental molecular questions pertaining to human endometrial biology.

## Data Availability Statement

The datasets presented in this study can be found in online repositories. The names of the repository/repositories and accession numbers can be found in the article/Supplementary Material.

## Ethics Statement

The studies involving human participants were reviewed and approved by Monash Health and Monash University Human Research Ethics committees. The patients/participants provided their written informed consent to participate in this study.

## Author Contributions

RL: collection and assembly of data, data analysis and interpretation, manuscript writing, and final approval of the manuscript. PV: data analysis and interpretation, final approval of the manuscript. SG: data analysis and interpretation, manuscript revision and final approval of manuscript. CF: collection and assembly of data, data analysis and interpretation, manuscript writing, and final approval of manuscript. SD: collection and assembly of data, data analysis and interpretation, and final approval of the manuscript. JM: data analysis and interpretation, manuscript editing, and final approval of the manuscript. SO: data analysis and interpretation, final approval of manuscript. JB: conception and design, data analysis and interpretation, manuscript editing, and final approval of manuscript. CG: conception and design, financial support, data analysis and interpretation, manuscript editing, and final approval of manuscript. All authors contributed to the article and approved the submitted version.

## Conflict of Interest

The authors declare that the research was conducted in the absence of any commercial or financial relationships that could be construed as a potential conflict of interest.
