# Supplementary material for: Impact of Sustained Transforming Growth Factor-β Receptor Inhibition on Chromatin Accessibility and Gene Expression in Cultured Human Endometrial MSC
Source: Front Cell Dev Biol. 2020 Sep 1;8:567610. doi: 10.3389/fcell.2020.567610 (PMC7490520; doi:10.3389/fcell.2020.567610)
Supplement: Supplementary file 1 [file Data_Sheet_1.pdf]

Supplementary Material

Impact of Sustained Transforming Growth Factor-β Receptor Inhibition on Chromatin Accessibility and Gene Expression in Cultured Human Endometrial MSC

Raffaella Lucciola[1,2,3], Pavle Vrljicak[3,4], Shanti Gurung[1,5], Caitlin Filby[1,2], Saeedeh Darzi[1,2], Joanne Muter[3,4], Sascha Ott[3,4], Jan J. Brosens[3,4] and Caroline E. Gargett[1,2*]

*** Correspondence:**

Caroline E. Gargett

caroline.gargett@hudson.org.au

Table S1 Regulation of pathways preventing cellular senescence in A83-01-treated eMSC

	Category	Term	Genes	Fold Enrichment*
**Up-regulated genes**	GOTERM_MF_DIRECT	peroxidase activity	CYGB, GPX3, PTGS2	3.74
	GOTERM_BP_DIRECT	response to oxidative stress	CYGB, GPX3, PTGS2, SCARA3, APOE, DUSP1	1.46
	GOTERM_BP_DIRECT	cellular oxidant detoxification	CYGB, GPX3, PTGS2, APOE	1.53
**Down-regulated genes**	KEGG_PATHWAY	p53 signaling pathway	CDK6, CCND2, THBS1, ATM, TP53I3, IGF1, CDKN1A, RPRM, PTEN	3.82
	BIOCARTA	cyclins and cell cycle regulation	CDK6, CCND2, CDKN2B, CDKN1A	4.41
	BBID	cyclin-CDK complexes	CDK6, CDKN2B, CDKN1A	5.97
	GOTERM_CC_DIRECT	cyclin-dependent protein kinase holoenzyme complex	CDK6, CCND2, CDKN1A	6.04
	BIOCARTA	Cell Cycle: G1/S Check Point	CDK6, ATM, CDKN2B, CDKN1A	3.67
	KEGG_PATHWAY	cell cycle	CDK6, CCND2, ATM, CDKN2B, CDKN1A	1.15

***** Fold enrichment represents enrichment of the indicated GO term in A83-01 treated versus untreated samples.

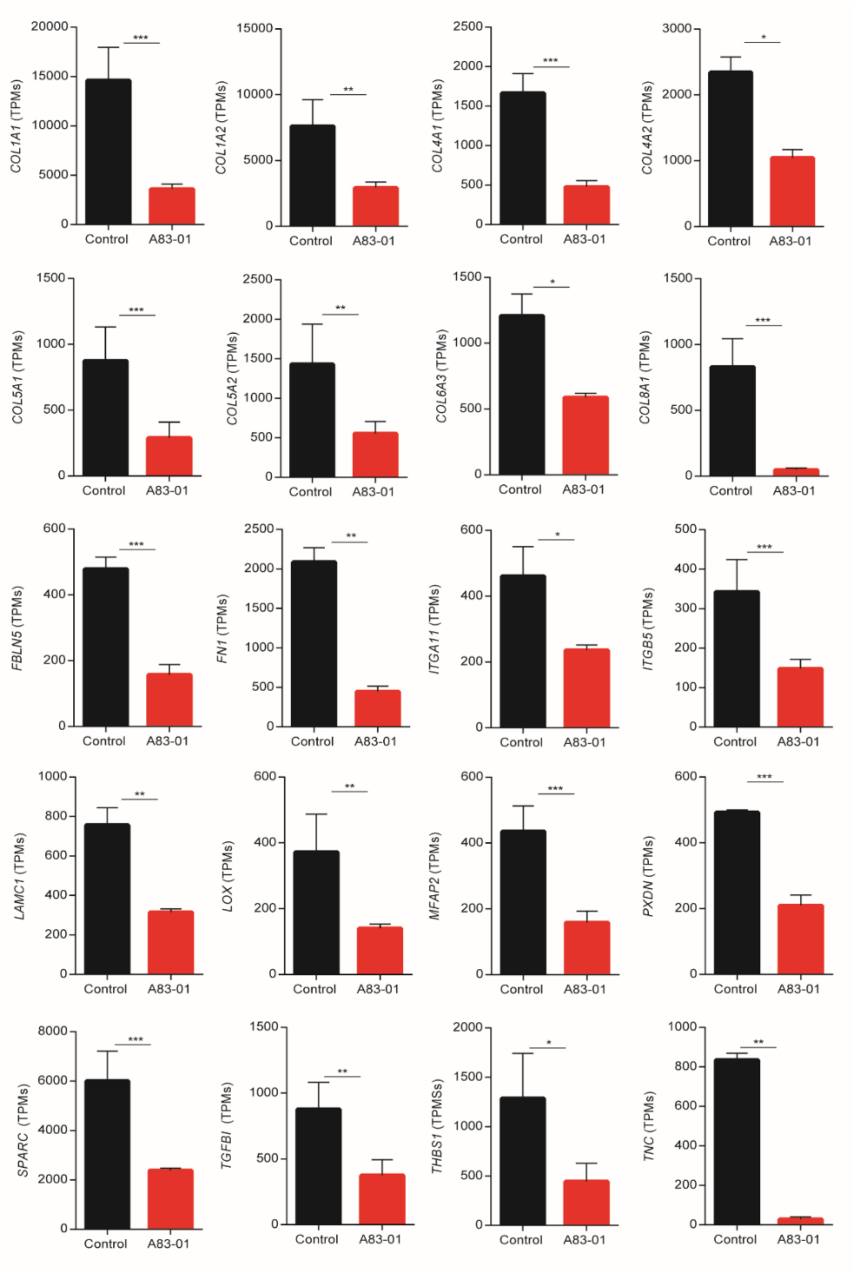

Figure S1. ECM genes down-regulated in response to A83-01 treatment. Graphs showings changes in level of gene expression of the top 16 most abundant ECM genes negatively regulated by TGFβ-R inhibition, represented as changes in TPMs. Data represent mean ± SEM; Y-axis shows TPMs; *indicates $q < 0.05$, ** $q < 0.01$ and *** $q < 0.001$.

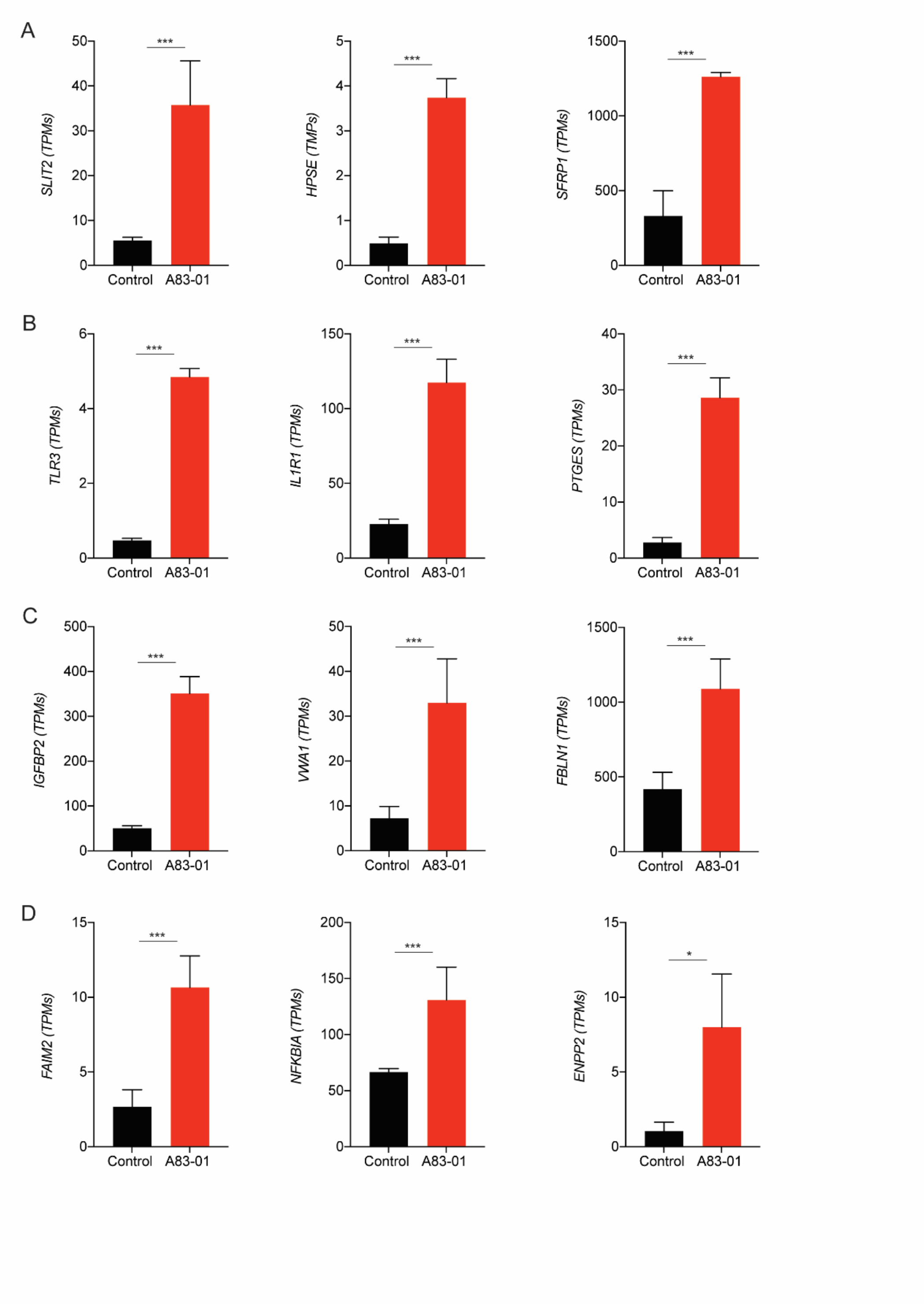

Figure S2. Examples of angiogenic (A) anti-inflammatory, (B) immunomodulatory (C) antifibrotic and (D) antiapoptotic genes up-regulated in response to A83-01 treatment. Graph show changes in gene expression level (TPMs) of positively regulated genes by TGFβ-R inhibition. Data represent mean ± SEM; Y-axis shows TPMs; * indicates q < 0.05, ** q < 0.01 and *** q < 0.001.

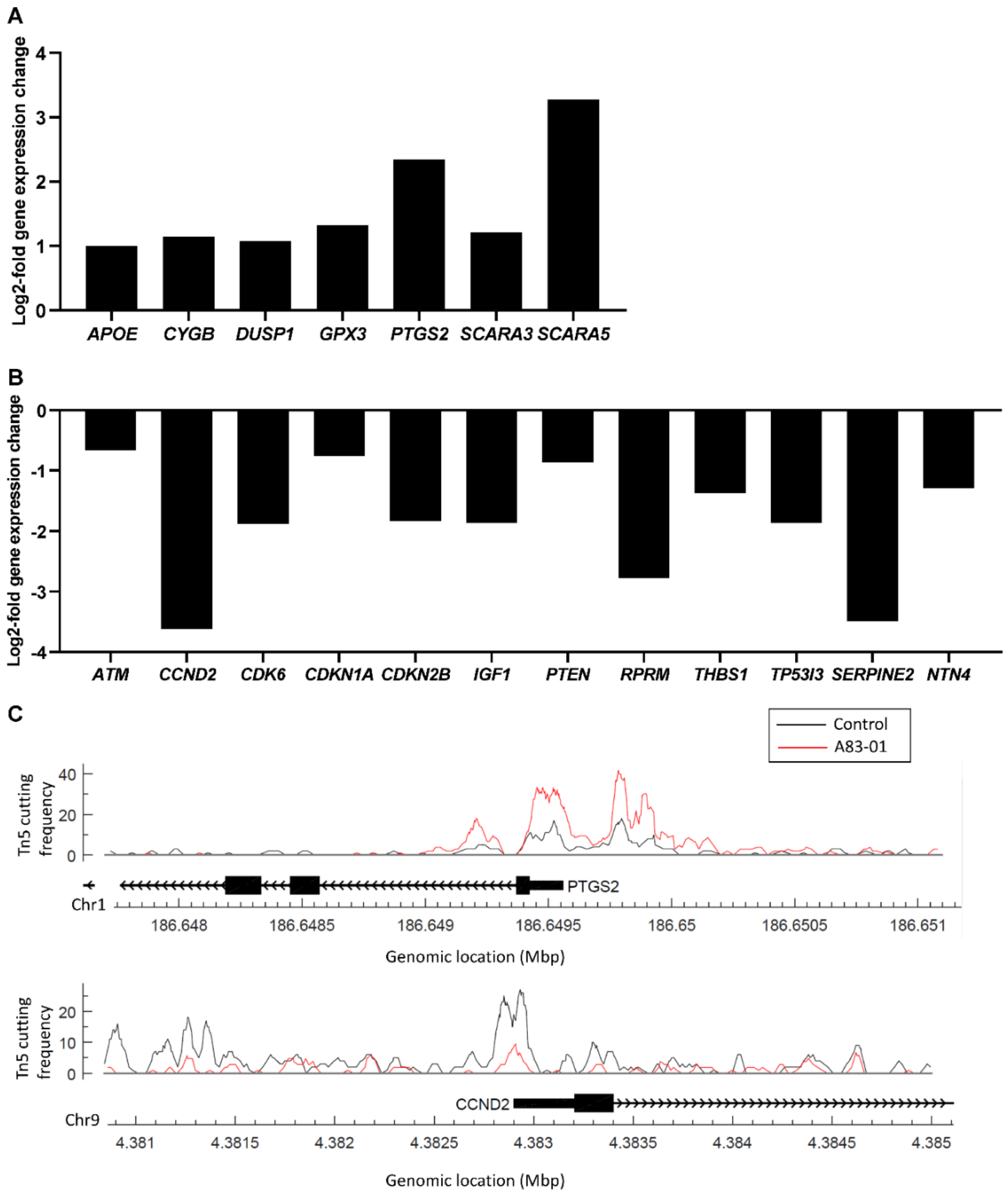

Figure S3. Regulation of senescence related genes. A83-01 prevents eMSC senescence through **A)** induction of genes implicated in preventing oxidative damage and **B)** inhibition of cellular senescence-related genes. **C)** ATAC peak showing opening and closing of the chromatin accessibility downstream of the promoter region of *PTGS2* and *CCND2*, respectively. Black and red traces represent untreated and A83-01-treated eMSC. The X-axis shows the genomic location of the ATAC-seq peaks and genes.

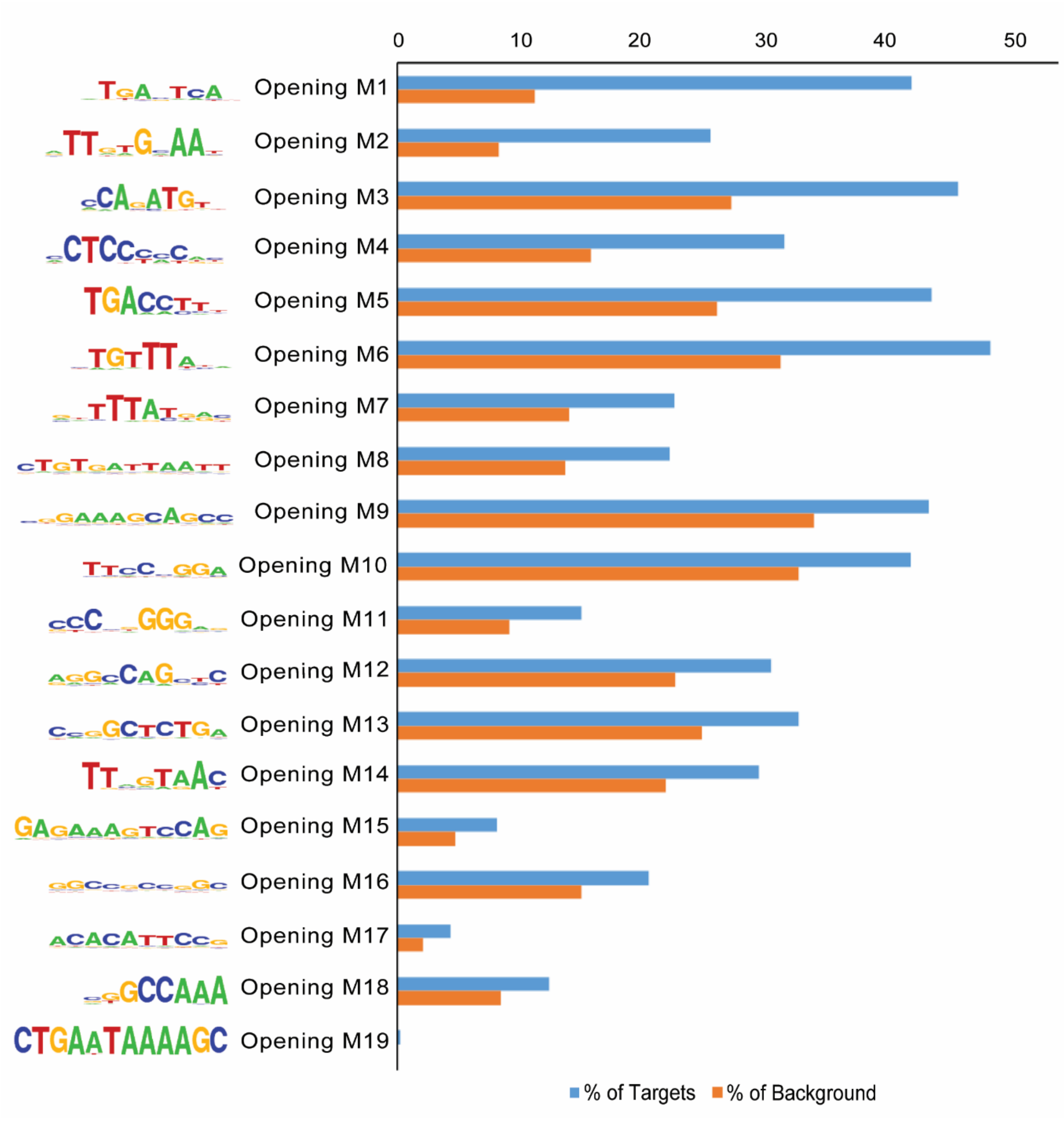

Figure S4. Enrichment of TF binding motifs in opening genomic regions. Total of 19 TF binding motifs enriched in the opening ATAC-seq peaks. The frequency (%) of peaks (blue bars) containing a given motif is shown relative to genomic regions randomly selected from the genome (orange bars) (±50 kb from TSS, matching size, and GC/CpG content.

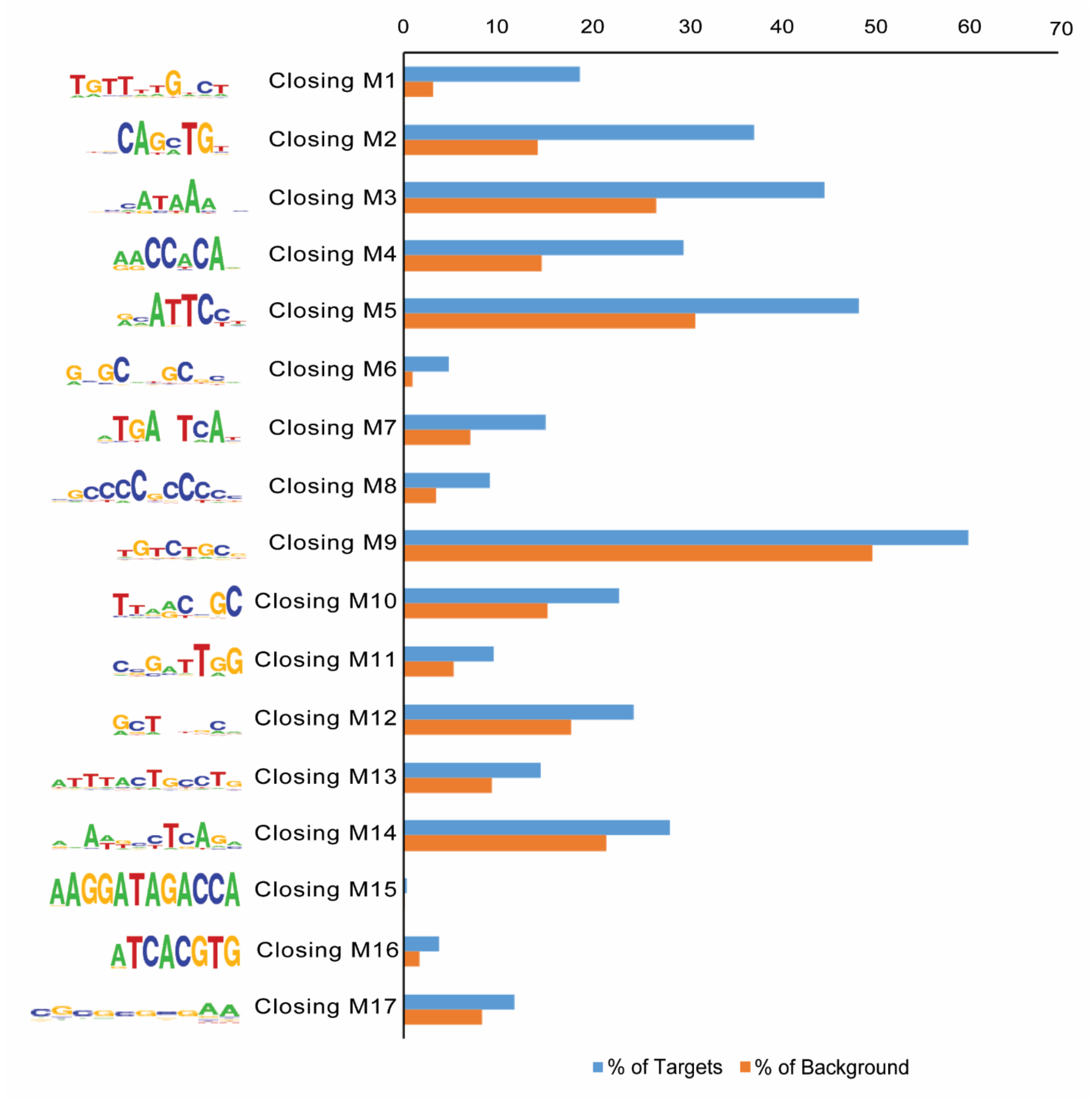

Figure S5. Depletion of TF binding motifs in closing genomic regions. Total of 17 TF binding motifs enriched in the closing ATAC-seq peaks. The frequency (%) of peaks (blue bars) containing a given motif is shown relative to genomic regions randomly selected from the genome (orange bars) (±50 Kb from TSS, matching size, and GC/CpG content).
